# The Etiology of Elephantiasis, (Grecorum and Arabum,) Endemic Hæmaturia and Chyluria

**Published:** 1876-03

**Authors:** Chas. H. Richmond

**Affiliations:** Livonia, N. Y.


BUFFALO

### Medical and Surgical Journal.

VOL XV. MARCH, 1876. No. 8.

### Original Communications.

:o:

ART. I.—*The Etiology of Elephantiasis* (*Grecorum and Arabum,*) *Endemic Hœmaturia and Chyluria.* By CHAS. H. RICHMOND, M. D., Livonia, N. Y.

[Extract from an Address on Sanitary Science, read before the Alumni Associaion of the Medical Department, University of Buffalo, Feb. 23, 1876.]

I wish to call attention to the etiology of certain diseases of common occurrence in tropical countries, in order that, by fully appreciating their real nature, they may at least be held in abeyance and be kept from more frequent observation upon our shores. I refer to the elephantoid diseases, Egyptian and Brazilian hæmaturia (bloody urine) and chyluria (milky urine).

In the investigation of this subject, I shall refer especially to the experience of Mr. T. B. Lewis, of the British military service in India, concerning the pathological significance of microscopic worms inhabiting the blood. I gave a synopsis of his discoveries in a review of his book* in the *N. Y. Medical Journal* for November, 1875, and I will now briefly state some of the points elicited by his investigations.

Lewis examined the blood of a large number of pariah dogs of

* "The Pathologicaal Significance of Nematode Hæmatozoa." By T. B. Lewis, M. B., Calcutia. 1874.

India, and discovered in about one-third the whole number experimented on *filariæ*, or minute blood-worms, resembling the *filariæ sanginis hominis*, but which presents some slight points of difference in microscopical appearance. The nematode hæmatozoa occasionally found in man is about the same size as those found in the dog, namely: averaging 1-75th of an inch in length by 1-3500th in diameter. When first hatched, their size is much smaller. Lewis is of the opinion that they belong to the class known as the *filariæ sanguinolenta*, which, under favorable circumstances in the blood of dogs, develop into thread-like worms of from two to six inches in length.

The behaviour of the parasites within the body of the dog is quite interesting to study. They are probably received into the system by being swallowed in their larval state, and becoming lodged in the walls of the œsophagus, undergo development and migrate into neighboring parts, the aorta being more frequently infested. Tumors, composed of the larvæ or minute worms already hatched, are formed in the walls of the œsophagus and aorta varying in size from that of shot to a walnut. The blood does not seem favorable to the hatching of the eggs when deposited in that fluid, but it is favorable to their growth after they hatch and begin to develop in the tissues. It is not known that in man filariæ ever become so largely developed as do the hæmatozoa in the dog, but they migrate throughout all the tissues in the same remarkable manner as in the case of the wild dogs of India. It may be well to state that these parasites do not resemble, either in their anatomical formation or behavior within the body, the cysticircæ, trichinæ, or the echinococci.

So far as observations have extended respecting the salient phenomena produced by the migration of these nematoda in man, the fact is elicited that the principal are the escape of the nutritive fluids of the body from their proper channels—either the chylous or sanguineous fluids, or an obstruction to their flow. The escape may be either into the subcutaneous tissues or into an execretory duct. When the chyle is impeded in its passage, elephantiasis arabum (elephant leg) may result. Virchow and

Rindfleisch* both attribute this disease to obstruction of the lymphatics ; if it escape into tissue sufficiently to interfere with the nutrition of the part or to induce ulceration, elephantiasis gre_ corum, or true leprosy is developed; if, however, the escape is into an excretory duct, as, for instance, into the ureter, chyluria is produced ; when bloodvessels are perforated, sanguineous discharges or deposits may occur.

These pathological conditions, which have until recently been unexplained, have been found associated with the presence of nematode hæmatozoa in a sufficient number of instances, by different observers, to establish with a strong degree of probability a causative relation on the part of the latter. In one case has a microscopic nematode of a new species been found by Dr. Sonsius in the blood of a person suffering from Egyptian hæmaturia†; Dr. Wacherer has discovered a microscopic nematode in the urine of a person affected with Brazilian hæmaturia, and in no less than thirty persons with chyluria or elephantoid disease, or both, has Lewis found the parasite in the blood, excretions or tissues. It is not ascertained positively that the hæmatozoa found by the different experimenters are the same in nature. This is a point for future observations to determine.

In the case of *leprosy* all *facts*, so far as observation has extended in connection with its clinical history and pathological anatomy, are entirely compatible with the theory that the deposits are occasioned by a leakage of the chyle into the affected tissues.

1. It may stated that no age or clime is exempt from the disease, while in India and other tropical countries it is principally observed at the present time, within the past few centuries it has existed in Europe and America as far north as Norway and New Brunswick. At one time it prevailed as extensively in Norway as it ever has in any part of the globe.‡

2. It seems to occur irrespective of the kind of food employed or of the habits of life. The idea that it was produced by eating largely of fish, which at one time prevailed, is thought by Tilbury,

---

* *Vide* "Erichsen's Science and Art of Surgery." 1873.

† In his "Research concerning the Bilharzia Hæmitobia in relation to the Endemic Hæmaturia of Egypt," he bases his opinion upon an analysis of ten cases that that disease is due to the parasites.

‡ *Vide* "Aitken's Science and Practice of Medicine." Philadelphia, 1872.

Fox, H. Vandyke Carter, Farquhar, and other observers of leprosy, to have but very little, if any, influence in its production. Any single article of food long continued, or any diet which does not contain the necessary elements for the proper nutrition of the tissues, must exert some influence in the course of leprosy; but it cannot be shown to cause it *de novo.*

3. Microscopical and chemical examination of the deposits in elephantiasis shows that they correspond with the nutritive fluids of the body, influenced only by such changes as would be likely to be induced by admixture with inflammatory products, and by certain changes in the tissues themselves.

4. It is a disease propagated by inoculation.

Tilbury Fox, in his treatise on "Skin Diseases,"* includes four causes in the propagation of leprosy: 1. Intermarriage of lepers; 2. Hereditary transmission; 3. Inoculation and cohabitation; and 4. Vaccination (to a slight extent.)

In discussing these factors in turn, we may well inquire if they do not all resolve themselves into those induced by the ingestion of food or drink containing the elements of the disease, and inoculation. How far hereditary predisposition alone may influence the propagation of the disease is uncertain. It is true that a large number of children of leprous parents at some time during their life show signs of elephantiasis in some form, or milky urine; yet, with the numerous opportunities for the contact of children with their parents, it may fairly be questioned whether the supposed hereditary influence may not really be an inoculation. In known instances of infection by intermarriage, cohabitation and vaccination, inoculation of a *special element* in its production, whatever it be, is the only explanation tenable.

Assuming filariæ to be the sole element in the production of these affections, we have a reasonable explanation of all the phenomena observed in their etiology—that is, every pathological procsss observed in these diseases might readily be induced by migratory parasites, were they known to exist in the blood; furthermore, the known means of propagation of filariæ, as studied

† 2d Am. Ed., p. 320, *et seq.*

in the dog, is in accordance with the theory of inoculation and ingestion.

I am aware that perhaps the majority of writers do not recognize any pathological connection between elephantiasis grecorum and elepantiasis arabum, nor between these affections and chyluria; but when we remember that these affections occur in the same localities at the same time, and sometimes in the same individual at the same or at different times during his life, support will be lent to their close relationship, if not to their identity, as regards etiology.

The reasons why the same special cause affects different individuals in ways so diverse, are demonstrable, although it may be somewhat surprising that such diversities should exist. In the case of dogs affected with hæmatozoa, according to Lewis, some present a most miserable condition, while others exhibit no apparent deviation from health; hence it is to be inferred that the mere presence of hæmatozoa, within certain limits, in the blood may produce no noticeable effects. It is only when they exist in sufficient numbers to interfere with the capillary circulation, and when they migrate to other parts, that their effects are discernable. Possibly the filariæ is a native of the dog, so that less injury is induced in that animal than in man, on the same principal that trichinæ are innocuous in the rat and pig, but dangerous to man. In man the circulation of the blood and chyle may be impeded by tumors formed by the parasites, either in their larval state or while undergoing development; thus, if such stoppage occur in the kidney, hæmaturia may occur as the result of blood pressure; if a stoppage of the lymphatics of the thigh occur sufficiently to cause inflammation of those vessels, so a greater accumulation of lymph occurs than can undergo absorption, a rational explanation of the occurrence of Barbadoes leg is found.

An explanation of the frequent infection of the lymphatic system is found in the close anatomical relation borne between the aorta and the receptaculum chyli and principal lymphatics. The nearness of these lymphatic vessels to the ureters will account for a direct transfer of the chyle into those organs with milky urine if migration of the worms takes place in points favorable to such transmission. Chyluria is usually temporary, with a liability to return, while the anatomical changes in elephantiasis of the leg, scrotum, etc, are usually permanent. In true leprosy, in addition to the change induced by the escape of chyle into the subcutaneous tissues, and a bad hygienic condition which usually coexists, the nervous system becomes affected, probably either by direct attack of the worm or from encroachments of a nematoid tumor. Carter has found the nerves of affected parts in the anæsthetic form swollen and red in some instances, and translucent and firm in others. The infiltration or deposit surrounds the nerve-tubules, affecting the funiculus itself rather than the connective coat of the nerve. He thinks the affection of the nerves takes its origin in the peripheral branches and extends towards the central system. Both Danielssen and Bock, on the other hand, contend that the primary seat of the nervous complication is in the spinal cord, as they have found the cord and its membranes infiltrated with an albuminous deposit, the cord being indurated and discolored. They have also found the sheaths of certain nerve branches and ganglia similarly affected.* In the tubercular form of leprosy, the deposit is found in the fibro-cellular structures throughout the body.

Thus it will be understood why there may be so much diversity in the anatomical appearances occasioned by the presence of nematode in the system. But more than the one condition may coexist, as has been stated. Lewis cites cases of chyluria which occurred in lepers of several years' standing, and in other forms of elephantoid disease, filariæ having been detected in both the blood and urine of the patients. Ewart, Coull MacKenzie, McConnell and McLeod,* have each had an example.

Although Lewis is the most extensive investigator of the subject, others have previously suggested the possible occurrence of elephantoid disease by the agency of parasites in the circulation. Fayrer, an extensive observer, makes the suggestion in his "Clinical and Pathological Observations in India," published in London in 1873 ; and Palmer, in the *Indian Med. Gazette*, vol. viii., 1873, advances a similar supposition.

* T. Fox, *log. cit.*

† *Indian Med. Gazette*, Aug., 1874.

The failure to demonstrate the facts herein stated until recently may beget doubts in the minds of some as to the verity of conclusions; however, when we consider the difficulties experienced in discovering the minute parasite, thorough microscopical skill being required, it is scarcely a matter of surprise that the essential element in the etiology of these affections has hitherto remained undiscovered. Besides, the parasite need not necessarily exist in deposits in case of elephantiasis, so that study of the morbid anatomy alone would tend to conceal the intimate factor. It seems, moreover, that the number of cases of these diseases in which the filariæ are found to be associated is sufficient to establish a relationship.

It is true that this question is now in its infancy, and it remains for future inquiry to complete and perfect our knowledge in this direction. Nor is it yet understood to what extent the diseases in question are preventable. We are thus led to consider, in a word, some of the practical questions to be deduced from these inquries.

I am not aware that any observer has demonstrated the tenacity with which the filariæ of the human blood, or that of the dog or the ova cling to life; and until that point is determined, we cannot tell how far it is safe to admit lepers and persons affected with chyluria or hæmaturia into society. It is safer to adhere to the ancient law of Moses, which required the complete banishment of that class from society; and in addition, to place them within circumscribed limits, in order that the territory upon which sound individuals reside may not be in any way infected. The passage of all excrementitious matters, urinary and fecal, should be so regulated as to render it impossible for any animal to gain access to them, and that any parasities possibly excreted, or their ova, should likewise fail of gaining access to either man or animals. Intermarriage should be peremptorily interdicted. It may seem a cruel procedure to separate families, and banish and forbid marriage to persons in comparatively good health, but who are subject to occasional attacks of milky or bloody urine, or who may be suffering from an elephant leg; but the future welfare of the human race warrants it for their protection, and it should be enforced.

It is better that a few in the present generation should suffer social ostracism than that the world hereafter suffer in consequence of the unnecessary propagation of some of the most loathsome affections known to man. All persons thus banished should receive good hospital treatment, and by cleanliness and the ordinary comforts of life, be made as happy as possible. As it is a question still open whether these affections are transmitted by hereditary influence, or by contact incident the filial relation, prevention of cohabitation or marriage with a leper cannot be insisted on too strongly, inasmuch as it is far from desirable to increase the number of that unfortunate class.

Wild dogs, in countries in which these diseases are rife, should be indiscriminately slaughtered and burned, or buried out of reach of any animal likely to feed upon them. Dogs were and still are regarded by the Orientals with abhorrence; and if we may rely upon the statements made in this paper, we should say, with much justice, all animals which, by future experiments, are found to contain nematoda, should be interdicted as food, and care should be used in selecting grain for flour, and be sure that it is free from smut.

The laws of uncleanness of the Jews, without doubt, had a purpose, and if understood in all their bearings, might, for the most part, with propriety be enforced at the present time. The prevention of the class of diseases herein discussed may properly be agitated in our country, for we know not how soon we shall be required to practice all the knowledge we may gain upon the subject.

It is for this reason that I thought it not altogether impractical for your consideration on this occasion. Should future research confirm the essential points herein advocated, we may feel that no time has been lost in a partial attempt to familiarize ourselves with this subject.

